# Dietary Fatty Acid Saturation Modulates Sphingosine-1-Phosphate-Mediated Vascular Function

**DOI:** 10.1155/2019/2354274

**Published:** 2019-08-25

**Authors:** Daniel W. Nuno, Kathryn G. Lamping

**Affiliations:** ^1^Department of Internal Medicine, Roy J. and Lucille A. Carver College of Medicine, University of Iowa, Iowa City, IA, USA; ^2^Iowa City Veterans Affairs Healthcare System, Iowa City, IA, USA; ^3^Department of Pharmacology, Roy J. and Lucille A. Carver College of Medicine, University of Iowa, Iowa City, IA, USA

## Abstract

Sphingolipids, modified by dietary fatty acids, are integral components of plasma membrane and caveolae that are also vasoactive compounds. We hypothesized that dietary fatty acid saturation affects vasoconstriction to sphingosine-1-phosphate (S1P) through caveolar regulation of rho kinase. Wild type (WT) and caveolin-1-deficient (cav-1 KO) mice which lack vascular caveolae were fed a low-fat diet (LF), 60% high-saturated fat diet (lard, HF), or 60% fat diet with equal amounts of lard and n-3 polyunsaturated menhaden oil (MO). Weight gain of WT on HF and MO diets was similar while markedly blunted in cav-1 KO. Neither high-fat diet affected the expression of cav-1, rho, or rho kinase in arteries from WT. In cav-1 KO, MO increased the vascular expression of rho but had no effect on rho kinase. HF had no effect on rho or rho kinase expression in cav-1 KO. S1P produced a concentration-dependent constriction of gracilis arteries from WT on LF that was reduced with HF and restored to normal with MO. Constriction to S1P was reduced in cav-1 KO and no longer affected by a high-saturated fat diet. Inhibition of rho kinase which reduced constriction to PE independent of diet in arteries from WT and cav-1 KO only reduced constriction to S1P in arteries from WT fed MO. The data suggest that dietary fatty acids modify vascular responses to S1P by a caveolar-dependent mechanism which is enhanced by dietary n-3 polyunsaturated fats.

## 1. Introduction

Poor diet composition is a major contributor to the epidemic of overweight and obese individuals who are at an increased risk of developing type 2 diabetes and cardiovascular disease. Excess fat consumption leads to weight gain and dyslipidemia. While abnormal levels of triglycerides, free fatty acids, and cholesterol contribute to dyslipidemia, other lipid species are generated with high-fat diets which could contribute to development of vascular disease. Sphingolipids are a complex class of lipids incorporated into cell membranes that also contribute to cell signaling. The sphingolipid ceramide produced in all tissues is elevated with a high-saturated fat diet, hypertension, type 2 diabetes, and insulin resistance [1–6]. The impact of elevated ceramide levels is broad ranging since ceramide affects the activity of many kinases, phosphatases, transcription factors, and even vasculature [7, 8]. Ceramide accumulates within cells where it is metabolized to other sphingolipids. Therefore, any dietary manipulation that alters the levels of ceramide will also affect the levels of other sphingolipid derivatives. Sphingosine-1-phosphate (S1P), a ceramide metabolite, is generated from deacylation of ceramide by ceramidases to form sphingosine and subsequent phosphorylation by sphingosine kinase 1 or 2 to form S1P. Levels of S1P are increased in genetic and diet-induced animal models of obesity and humans with obesity, atherosclerosis, and cardiovascular disease and can also affect vascular function [9–13]. The impact of dietary fatty acids differing in the saturation level on S1P and vascular function has not been addressed.

Although there is a consensus that reducing dietary saturated fats is a key for preventing or reversing the development of obesity, the appropriate substitute for saturated fats is controversial. Diets enriched in either monounsaturated (MUFA) or polyunsaturated fatty acids (PUFA) improve cholesterol levels and insulin sensitivity compared to diets enriched in saturated fats, but the benefits of MUFAs versus PUFAs in preventing or reversing cardiovascular disease have not been consistent [14, 15]. Long chain PUFAs categorized into n-3 and n-6 fatty acids based on the position of the double bond are incorporated into the plasma membrane affecting membrane order and function. In addition to affecting membrane structure, dietary fatty acids differing in saturation also impact lipid metabolism and formation of sphingolipids [16]. A high-saturated fat diet increases ceramide levels in the skeletal muscle and liver which is associated with adverse cardiovascular effects [1, 10, 16–18]. Substituting saturated fats with MUFA or PUFA may affect the generation of ceramide versus other sphingolipids and the development of adverse cardiovascular events.

We previously compared the effects of a high-saturated fat diet versus a mixed high-saturated fat and n-3 PUFA diet on vascular reactivity [19, 20]. Both high-fat diets produced similar degrees of obesity and reduced localization of endothelial nitric oxide synthase (eNOS) in caveolin-1- (cav-1-) containing lipid rafts. Reducing localization of eNOS within cav-1-containing lipid rafts enhances the activation of eNOS and release of nitric oxide which increases nitric oxide-mediated dilation [21–24]. However, in our study, only the n-3 PUFA-supplemented diet increased the dilation of arteries to acetylcholine compared to low fat [20]. Inhibition of NOS did not abolish the difference in acetylcholine-induced dilation in arteries from mice on the two high-fat diets [20]. These data suggest that dietary fats affect other mechanisms that modify vascular function. In this study, we examined the impact of dietary fatty acids on sphingolipid-mediated vascular function. We tested the hypothesis that dietary fatty acids impact vascular responses by altering S1P-mediated vascular function which could be regulated by cav-1-containing lipid rafts, caveolae.

## 2. Methods

### 2.1. Diet-Induced Obesity

The animal protocol complied with the Guiding Principles for Research Involving Animals and Human Beings and was approved by the Animal Care and Use Committee of the Iowa City Veterans Affairs Health Care System. Male C57BL/6J (wild type, WT) and cav-1 deficient (cav-1 KO, B6.Cg-*Cav1^tm1Mls^*/J, stock #007083) were obtained from Jackson Laboratories. Only male mice were used in these studies since several studies have shown that female mice are resistant to effects of high-fat diet [25–27]. At 9 weeks of age, mice were randomly assigned to a normal low-fat diet (LF, 13% kcal fat, Teklad #7001, Harlan Labs, Madison, WI) or high-saturated fat diet (HF, 60% fat from lard, Research Diets, D12492, New Brunswick, NJ). To determine whether a change in dietary fatty acids reverses the effects of HF diet, after 12 weeks of HF diet, random groups of HF mice were switched to a 60% high diet with half of the lard replaced with n-3 PUFA-enriched menhaden oil (MO, Research Diets, D10122003). The mice were maintained on diets for an additional of 6-10 weeks. In a subset of mice, fasting glucose levels were measured using glucose oxidase test strips and a handheld glucometer and body composition was measured (nuclear magnetic resonance, Bruker LF90II). Mice were euthanized with ketamine/xylazine (100/10 mg/kg, ip), weighed, and tissues and serum collected for measurement of vascular function and western immunoblots.

### 2.2. Assessment of Vascular Function

Following euthanasia, the gracilis muscle and aorta were removed and placed in cold modified Krebs buffer (mmol/l: NaCl 118, KCl 4.7, CaCl_2_ 2.5, MgSO_4_ 1.2, KH_2_PO_4_ 1.2, NaHCO_3_ 25, and glucose 5). Gracilis arteries (~75-175 *μ*m diameter) were isolated from muscle, cannulated onto glass micropipettes filled with physiological salt solution in an organ chamber, and secured with suture before being pressurized to 60 mmHg pressure. Warmed (37°C), oxygenated (20% O_2_, 5% CO_2_, and 75% N_2_) modified Krebs solution was continuously circulated through the chamber as the arteries equilibrated for 45-60 minutes before study. Vessel diameter was measured with video microscopy and an electronic dimension analyzer (Boeckeler Instruments, VIA-100). The viability of each artery was defined by a minimum of 50% constriction in response to 50 mM KCl. Concentration response curves to S1P (10 nM to 1 *μ*M), phenylephrine (PE, 10 nM to 10 *μ*M), and KCl (25 to 75 mM) were performed. To test the contribution from rho kinase, concentration response curves were repeated following inhibition of rho kinase with H1152 (1 *μ*M, Alexis Chemicals).

### 2.3. Western Immunoblots

Following removal of fat, aortas were flash frozen in liquid nitrogen. For the preparation of whole-cell lysates, 3-4 aortas were pooled, dounced in liquid nitrogen, and denaturing lysis buffer added (Tris-HCl 50 mM, EDTA 0.1 mM, EGTA 0.1 mM, SDS 0.1%, NP40 1%, deoxycholic acid 2.4 mM, protease, and phosphatase inhibitors). Samples were sonicated three times on ice, centrifuged, and protein concentration measured with the bicinchoninic acid method. Equal aliquots of whole-cell lysate protein were subjected to SDS PAGE analysis. After blocking, immunoblotting was performed using antibodies to cav-1 (1 : 500, BD Sciences, #610407), rho (1 : 100, BD Sciences, #610991), rho kinase 2 (Rock II, 1 : 500, BD Sciences, #610623), and *β*-actin (1 : 500, Sigma-Aldrich, #A2228). Following extensive washing, blots were incubated with secondary antibodies conjugated to horseradish peroxidase and analyzed using ImageJ (NIH). Protein expression was normalized to actin.

### 2.4. Statistical Analyses

Results were analyzed using PRISM and are presented as mean ± standard error of the mean. Body weight, composition, glucose, and western immunoblots were compared using one-way ANOVA followed by Tukey's multiple comparison test. Concentration response curves for vascular responses were compared using repeated-measures two-way ANOVA followed by Sidak's for multiple comparisons. Significance was defined as *p* < 0.05.

## 3. Results

### 3.1. Effect of Fatty Acid Diet on Body Weight, Body Composition, and Glucose in WT and cav-1 KO Mice

**Figure 1:**
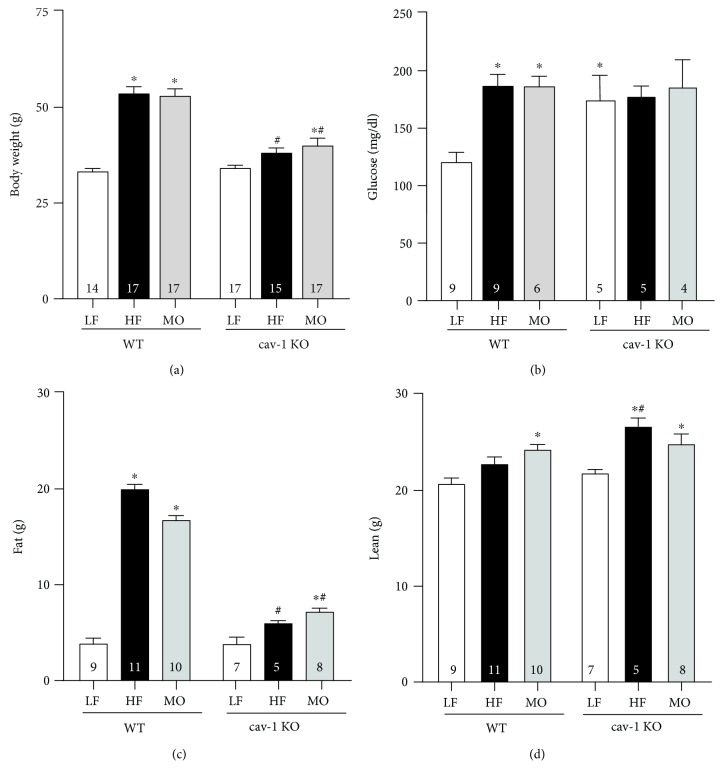
Body weight (a, g), fasting glucose (b, mg/dl), body fat (c, g), and lean mass (d, g) of WT and cav-1 KO mice on LF, HF, and MO diets. Mean ± SEM, *n* per group within each bar, ^∗^*p* < 0.05 vs. LF within the same genotype, ^#^*p* < 0.05 vs. WT mice on the same diet. ANOVA followed by Tukey's multiple comparison test.

The high-fat diet with saturated fat only and the high-fat diet with equal parts saturated fat and n-3 PUFA MO increased body weight in WT mice to the same extent (Figure 1(a)); however, the high-saturated fat diet increased body fat more than the MO-supplemented diet (HF vs. MO: *p* < 0.05; Figure 1(c)). The diet with MO tended to increase lean body mass in WT mice more than the saturated fat diet (Figure 1(d)). Body weight of cav-1 KO mice on normal LF diet was similar to WT but neither high-fat diet increased body weight of cav-1 KO to the levels of WT (Figure 1(a)). The inability of cav-1 KO mice to gain weight on the high-fat diets was due to little effect on body fat (Figure 1(c)). Surprisingly, both high-fat diets increased lean body mass (Figure 1(d)). Both high-fat diets increased fasting glucose levels in WT mice (Figure 1(b)). Fasting glucose levels were higher in cav-1 KO mice compared to WT irrespective of dietary fat (Figure 1(b)).

### 3.2. Effect of High-Fat Diet on Constriction to S1P in WT Mice

**Figure 2:**
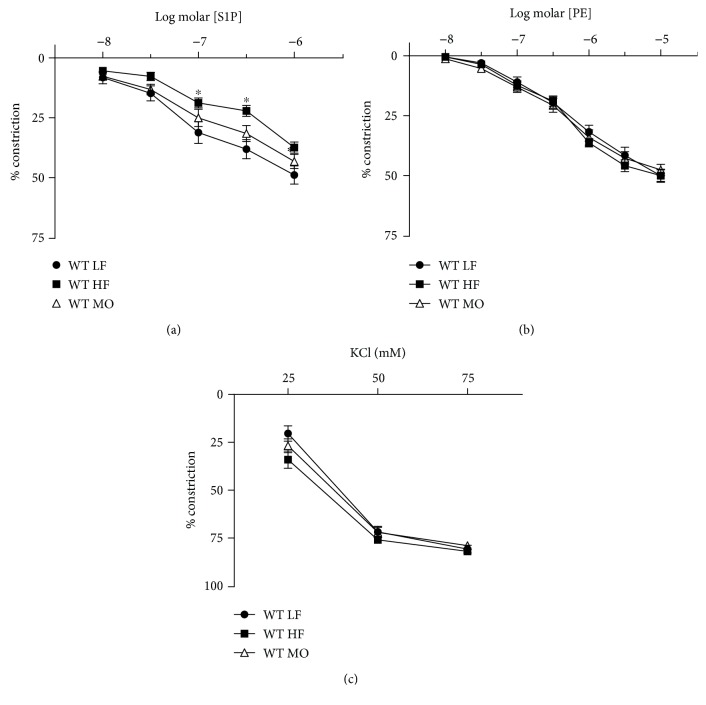
Constriction of gracilis arteries from WT mice on LF, HF, and MO diets to S1P (a), PE (b), and KCl (c). Mean ± SEM, *n* = 12‐16 per group, ^∗^*p* < 0.05 vs. LF. Two-way repeated-measures ANOVA followed by Sidak's multiple comparison test.

To determine the vasoactive properties of S1P, we assessed whether S1P constricted gracilis arteries under resting conditions or dilated constricted arteries. S1P failed to dilate gracilis arteries but produced a concentration-dependent constriction that was similar to PE (Figures 2(a) and 2(b)). Constriction to S1P was reduced in arteries from mice on HF diet but restored to normal when a portion of the saturated fat was replaced with MO (Figure 2(a)). In contrast to S1P, constriction to PE and KCl was not affected by either high-fat diet (Figures 2(b) and 2(c)).

**Figure 3:**
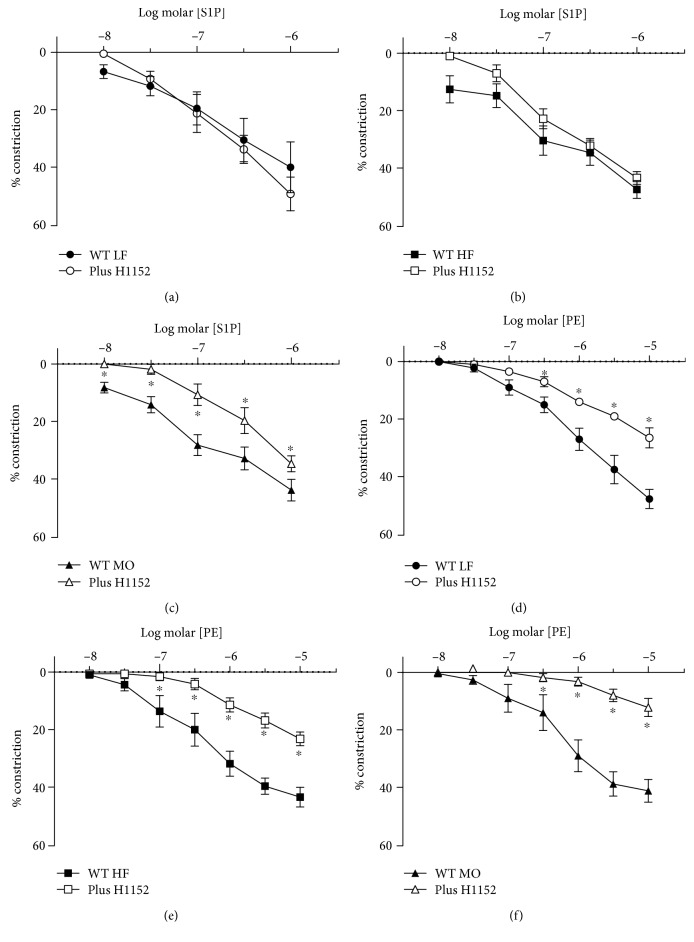
Constriction of gracilis arteries from WT mice on LF ((a, d), *n* = 4 each), HF ((b, e), *n* = 5 each), and MO ((c), *n* = 6 and (f), *n* = 5) diets to S1P (a-c) or PE (d-f) before and after inhibition of rho kinase with H1152 (1 *μ*M). Mean ± SEM, ^∗^*p* < 0.05 vs. without H1152. Two-way repeated-measures ANOVA followed by Sidak's multiple comparison test.

Our previous studies as well as others have demonstrated that constriction to many agonists is dependent on rho kinase [28, 29]. To determine whether constriction to S1P is, in part, mediated by rho kinase, we measured responses in the presence of the rho kinase inhibitor H1152 (1 *μ*M). Although H1152 reduced constriction to PE in arteries from WT mice on all three diets, it only reduced constriction to S1P in arteries from WT mice on MO (Figure 3). H1152 had no effect on constriction to S1P in arteries from WT mice on LF or HF (Figure 3). Thus, in contrast to PE, constriction to S1P was not mediated by rho kinase except in the presence of a diet enriched in n-3 PUFA.

### 3.3. Effect of High-Fat Diet on Constriction to S1P in cav-1 KO Mice

**Figure 4:**
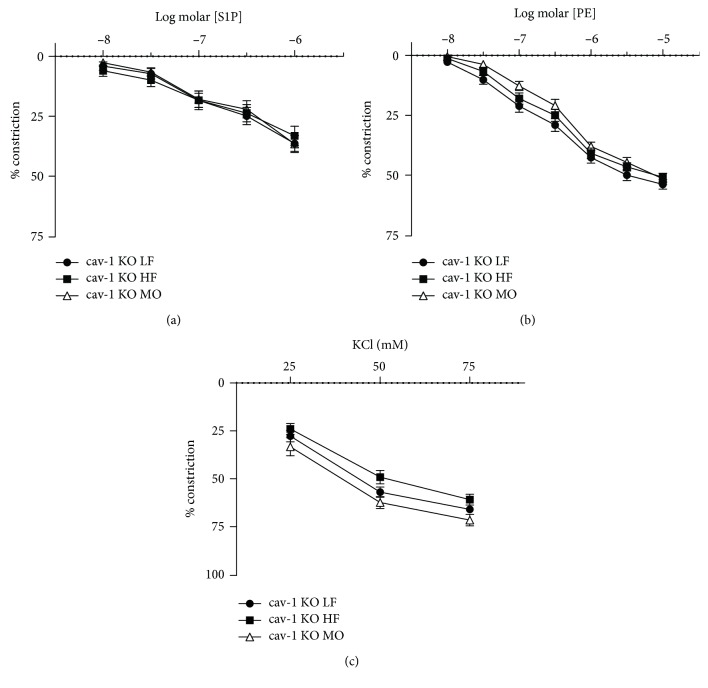
Constriction of gracilis arteries from cav-1 KO mice on LF, HF, and MO diets to S1P (a), PE (b), and KCl (c). Mean ± SEM, *n* = 12‐14 per group, ^∗^*p* < 0.05 vs. LF. Two-way repeated-measures ANOVA followed by Sidak's multiple comparison test.

**Figure 5:**
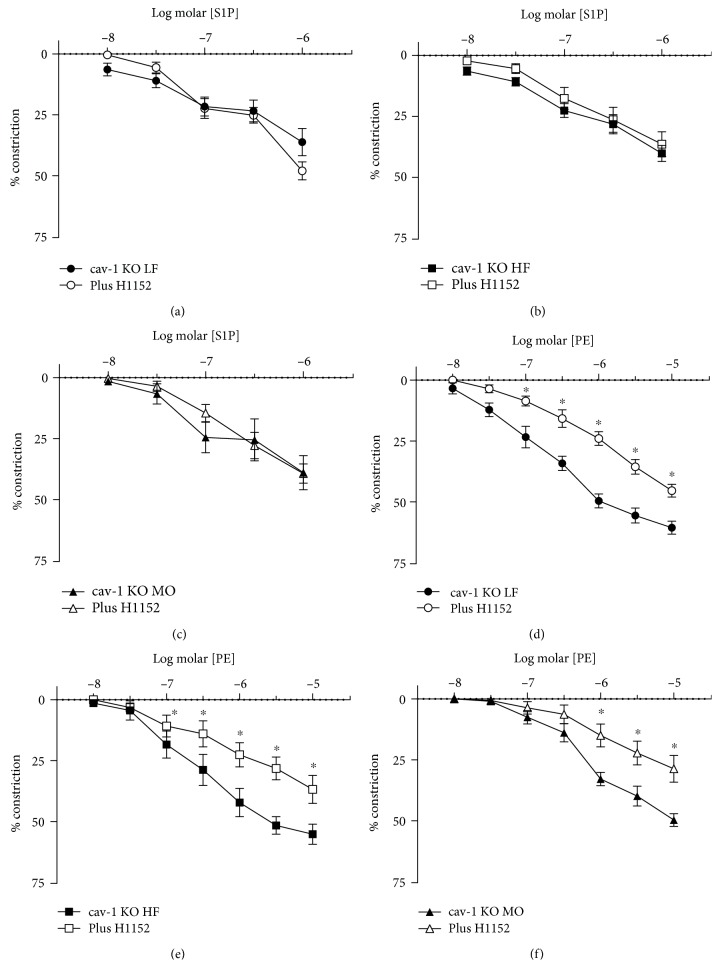
Constriction of gracilis arteries from cav-1 KO mice on LF ((a, d), *n* = 5 per group), HF ((b, e), *n* = 5 per group), and MO ((c, f), *n* = 6 per group) diets to S1P (a-c) or PE (d-f) before and after inhibition of rho kinase with H1152 (1 *μ*M). Mean ± SEM, ^∗^*p* < 0.05 vs. without H1152. Two-way repeated-measures ANOVA followed by Sidak's multiple comparison test.

We have previously demonstrated that the contribution from rho kinase in vascular constriction is modulated by cav-1 and caveolae [28]. To determine the role of caveolae in the effects of dietary fats on rho kinase-mediated responses to S1P, we examined responses to PE and S1P in arteries from cav-1 KO mice. Cav-1 is required for the formation of vascular caveolae which are absent in the vasculature of cav-1-deficient mice [21]. Constriction to S1P was reduced in arteries from cav-1 KO compared to WT (Figure 4(a)), and dietary fatty acid saturation no longer affected constriction to S1P in cav-1 KO mice (Figure 4(a)). Diet also had no effect on constriction to PE or KCl in cav-1 KO mice (Figures 4(b) and 4(c)). In contrast to WT mice, H1152 did not affect S1P-induced constriction in cav-1 KO mice on any diet while it still reduced constriction to PE on all diets (Figure 5). Thus, in the absence of cav-1 and caveolae, S1P-mediated constriction was reduced, dietary fatty acid composition no longer affected constriction to S1P, and inhibition of rho kinase did not reduce constriction to S1P in arteries from mice on MO. This effect of cav-1 was specific for S1P since inhibition of rho kinase still reduced constriction to PE.

### 3.4. Effect of High-Fat Diet on the Expression of Rho, Rho Kinase, and cav-1

**Figure 6:**
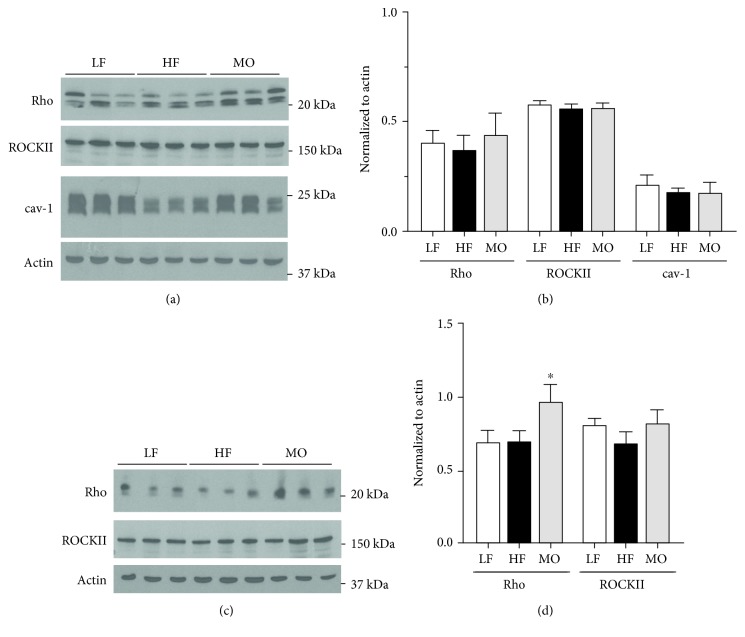
Representative and mean expression of rho, ROCK II, and cav-1 in the aorta from WT (a, b) cav-1 KO mice (c, d) on LF, HF, or MO diets. Mean ± SEM, *n* = 3‐9 per group, ^∗^*p* < 0.05 vs. LF, HF, or MO diet. ANOVA followed by Tukey's multiple comparison test.

To determine whether changes in protein expression could contribute to the effects of dietary fats on vascular responses, we compared the protein expression of rho, ROCKII, and cav-1 in arteries from WT and cav-1 KO mice on LF, HF, and MO diets. Neither high-fat diet significantly affected the expression of cav-1, rho, and ROCKII in arteries from WT mice (Figures 6(a) and 6(b)). In contrast to WT mice, dietary fats affected the protein expression in arteries from cav-1 KO mice. The diet with n-3 PUFA increased the expression of rho in the aorta but did not affect rho kinase (Figures 6(c) and 6(d)). High-saturated fat diet did not affect rho or ROCK II expression.

## 4. Discussion

Our previous studies and the present study comparing the effects of high-saturated fat diet versus mixed saturated and n-3 PUFA diets in mice have noted several differences. In our first study with short-term feeding WT mice a high-saturated fat diet versus a diet with the same amount of fat but equal amounts of saturated fat from lard and fish oil, weight gain was less in the fish oil-supplemented group compared to saturated fat diet only [19]. Extending the diet protocol for a longer duration eliminated the difference in body weight between WT mice on the two high-fat diets [20]. Both high-fat diets increased body weight and fasting glucose to the same level in WT mice [20]. Thus, differences in body weight or fasting glucose cannot account for the observed differences in vasoreactivity in arteries from mice on saturated fat and mixed saturated fat and n-3 PUFA. Weight gain in the cav-1-deficient mice on HF and MO was markedly less than in WT although fasting glucose was increased irrespective of diet [20]. Cav-1 deficiency and gene variants are associated with insulin resistance, diabetes, and the hallmarks of the metabolic syndrome [30–34]. We confirmed the dependence of glucose regulation on cav-1 reported by others [31, 35–38]. Cav-1-deficient mice were hyperglycemic even on a low-fat diet compared to WT. However, it should be noted that hyperglycemia in cav-1-deficient mice has not been reported in all cases [34]. Cav-1 within the endothelium may be crucial in defining insulin uptake into peripheral skeletal muscle where over 80% of glucose uptake occurs and is a potential site for development of insulin resistance [39, 40]. Thus, a high-fat diet increases body weight irrespective of the fatty acid composition. In the absence of cav-1 and caveolae, weight gain on a high-fat diet is diminished primarily because the high-fat diets do not increase body fat.

### 4.1. Sphingolipids and Vascular Function

The primary focus of these studies was to assess the impact of dietary fatty acid saturation on sphingolipid-mediated vascular function. Numerous studies have suggested that the sphingolipid ceramide affects vascular function. Acute ceramide exposure inhibited endothelium-dependent nitric oxide-mediated relaxation or enhanced contractions [41–45]. Ceramide itself is vasoactive since it relaxed rat aorta [46–48] but contracted other vessel types [49]. Inhibiting ceramide synthesis with myriocin prevented HF diet-induced ceramide accumulation and vascular dysfunction in WT mice [44]. In preliminary studies, we could not confirm any vasoactive effects of ceramide in the gracilis arteries of mice (data not shown). It neither dilated nor constricted gracilis arteries from WT mice on any diet. These contradictory effects of ceramide on vascular function may be related to differences in vessel size, tissue source, animal species, pathophysiology, or even generation of other ceramide metabolites.

The ceramide metabolite S1P is also reported to be vasoactive. A majority of studies report constriction to S1P although vasodilation has been observed [50–53]. S1P plays a role in myogenic tone of vasculature and the augmented myogenic tone in diabetes and hypertension [54–57]. Like ceramide, differences in the vasoactive properties of S1P may be related to species, tissue source, or vessel size-dependent properties. We initially tested the ability of S1P to dilate gracilis arteries in WT mice. S1P did not dilate submaximally contracted arteries from mice on any diet (data not shown) but produced a concentration-dependent constriction of gracilis arteries similar to reports of basilar arteries [52, 58]. Therefore, we focused on the effects of dietary fatty acid saturation on constriction to S1P in our study.

In WT mice, the S1P-mediated constriction of gracilis arteries was similar in magnitude and efficacy to PE. Constriction to S1P was reduced in arteries from mice fed the high-saturated fat diet compared to mice on a low-fat diet but was restored when a portion of the lard was replaced with n-3 PUFA-enriched menhaden. In contrast to PE, constriction to S1P was not dependent on rho kinase in arteries from mice on LF and HF diets. Surprisingly, inhibition of rho kinase only reduced constriction in arteries from WT mice on MO. In contrast to S1P, there was no effect of diet on the role of rho kinase in mediating constriction to PE. We have previously demonstrated a role for cav-1 and caveolae in rho kinase-dependent contractions of mouse aorta to serotonin [28, 29]. The diet-induced difference in S1P-mediated constriction and the contribution from rho kinase were not observed in arteries from cav-1 KO, while rho kinase was still involved in constriction to PE. The effect of dietary fatty acids on the rho kinase-dependent component was not related to changes in the expression of rho or rho kinase. Neither high-fat diets affected the expression of cav-1, rho, or ROCKII in WT mice. In contrast, in cav-1 KO mice, MO increased the expression of rho but not ROCK II which did not result in changes in vascular constriction. These findings suggest that the effects of the high-saturated fat diet on constriction to S1P are in part mediated by a cav-1 and/or caveolae-dependent mechanism. However, it is unlikely that the mechanism for the change in vascular responses with diet involves localization of rho or rho kinase in caveolae since we did not observe the same effects on rho kinase-mediated constriction to PE in arteries from WT and cav-1 KO mice. Rho kinase does not localize to caveolae [28], and in preliminary studies, we did not observe significant shifts in the localization of rho within lipid rafts of vascular tissue from mice on diets differing in saturated and unsaturated fats. These data suggest that other caveolar-regulated mechanisms may be affected by dietary fatty acid saturation that specifically impact S1P-mediated vasoconstriction. We have demonstrated that dietary fatty acids affect tissue fatty acid composition. A diet enriched in n-3 PUFA increases the levels of eicosapentaenoic and docosahexaenoic acids not only in the liver and skeletal muscle but also in vasculature [19, 20]. This change in vessel composition could impact the generation of vasoactive sphingolipids or caveolar localized signaling pathways involved in the S1P vasoreactivity. Thus, dietary fatty acid composition affected the vasoconstriction of peripheral small arteries to S1P through a caveolar-dependent mechanism that was independent of changes in the protein expression or localization of rho or rho kinase to lipid rafts.

## Data Availability

The data from these studies will be made available upon written request. Work described in this article was performed as part of the official duties of the authors to the federal government. Data release will require a specific request.
